# SWATH label-free proteomics analyses revealed the roles of oxidative stress and antioxidant defensing system in sclerotia formation of *Polyporus umbellatus*

**DOI:** 10.1038/srep41283

**Published:** 2017-01-30

**Authors:** Bing Li, Xiaofang Tian, Chunlan Wang, Xu Zeng, Yongmei Xing, Hong Ling, Wanqiang Yin, Lixia Tian, Zhixia Meng, Jihui Zhang, Shunxing Guo

**Affiliations:** 1 Institute of Medicinal Plant Development, Chinese Academy of Medical Sciences & Peking Union Medical College, Beijing 100193 P. R. China; 2 Pharmaceutical department of China-Japan Friendship Hospital, Beijing 100029 P. R. China; 3 Tianjin University of Science & Technology, Tianjin 300457, P. R. China; 4 State Key Laboratory of Microbial Resources, Institute of Microbiology, Chinese Academy of Sciences, Beijing 100101, P. R. China

## Abstract

Understanding the initiation and maturing mechanisms is important for rational manipulating sclerotia differentiation and growth from hypha of *Polyporus umbellatus*. Proteomes in *P. umbellatus* sclerotia and hyphae at initial, developmental and mature phases were studied. 1391 proteins were identified by nano-liquid chromatograph-mass spectrometry (LC-MS) in Data Dependant Acquisition mode, and 1234 proteins were quantified successfully by Sequential Window Acquisition of all THeoretical fragment ion spectra-MS (SWATH-MS) technology. There were 347 differentially expressed proteins (DEPs) in sclerotia at initial phase compared with those in hypha, and the DEP profiles were dynamically changing with sclerotia growth. Oxidative stress (OS) in sclerotia at initial phase was indicated by the repressed proteins of respiratory chain, tricarboxylic acid cycle and the activation of glycolysis/gluconeogenesis pathways were determined based on DEPs. The impact of glycolysis/gluconeogenesis on sclerotium induction was further verified by glycerol addition assays, in which 5% glycerol significantly increased sclerotial differentiation rate and biomass. It can be speculated that OS played essential roles in triggering sclerotia differentiation from hypha of *P. umbellatus*, whereas antioxidant activity associated with glycolysis is critical for sclerotia growth. These findings reveal a mechanism for sclerotial differentiation in *P. umbellatus*, which may also be applicable for other fungi.

Sclerotium is a special dormant form in the life cycle of fungi with compact hyphae and dehydrated outer coating in favor of its survival from extreme environment, but the precise mechanism of sclerotia differentiation from hyphae remains obscure. Many species of fungi can form sclerotia. Some of them can cause serious plant diseases, such as *Claviceps purpurea, Rhizoctonia solani, Sclerotinia sclerotiorum*^1^,^2^,^3^, whereas some are valuable food and medicine resources, such as *Ophiocordyceps sinensis*^4^. The unique coating structure confers sclerotia enhanced survivability against stressful conditions and resistance to antibiotics. *S. sclerotiorum* has ever been devastating plant pathogen and is difficult to control. *Polyporus umbellatus* (also named *Grifola Umbellata* or Zhuling) is a kind of traditional Chinese edible and medicinal fungus, and its sclerotia are used as diuretic drug in edema treatment and adjuvant in antitumor therapy. Its application is impeded because of resources exhaustion and germplasm degeneration. Understanding the initiation and maturing mechanisms is important for rational manipulating sclerotia differentiation, which would be beneficial for the revival of medicinal fungi resources, as well as for fungal pathogen control.

Various factors, such as physical conditions (low temperature, pH value etc), chemical reagents (fructose, glycerol etc) and biotic community (*Armillaria mellea* and companion fungus) can affect sclerotia differentiation individually or in combination. Reactive oxygen species (ROS) and oxidative stress (OS) are believed to be the key inducers of sclerotia formation upon stimulation of external factors, such as starvation, temperature variation and ionizing radiation. Georgiou^5^,^6^,^7^ looked into the mechanism of differentiation and growth of *S. sclerotiorum* sclerotia, and found that ROS were directly related to fungal cell differentiation^8^. Some ROS, such as hydrogen peroxide (H_2_O_2_), superoxide anion (O_2_^.−^) and hydroxyl radical (·OH) were detected in hyphae of *P. umbellatus*, and the relationship between ROS generation and sclerotia formation was established^9^,^10^. Sclerotia could not be formed at natural conditions on solid medium, and its initiation was associated with intracellular ROS accumulation. Antioxidants (diphenyleneiodonium, DPI) eliminating ROS could suppress sclerotia formation and caused biomass reduction via inhibiting reduced nicotinamide adenine dinucleotide phosphate (NADPH) oxidase and superoxide dismutase (SOD) to impair H_2_O_2_ generation^10^. Another experiment indicated that low temperature shift enhanced H_2_O_2_ generation in hypha cell wall or around the organelle membranes, and consequently induced *P. umbellatus* sclerotial formation^11^. These findings demonstrated that OS plays essential roles in sclerotia formation. However, the exact mechanisms of how sclerotia were induced by OS and how the cells survive from oxidative stress are unknown.

Mass spectrometry (MS)-based proteomics is becoming increasingly useful for qualitative and quantitative measurements of large numbers of complex protein samples in cells, tissues or organisms^12^. It has been applied in engineering filamentous fungi and other pathogens of human and plants^13^ in discovering regulatory circuits governing the fungal stress response^14^. Among the various MS technologies, label-free quantitative proteomics based on Sequential Window Acquisition of all THeoretical fragment ion spectra (SWATH)-MS provides good reproducibility, accuracy and precision in quantification of proteins, and is suitable for detecting negligible protein differentiation (less than two folds)^15^,^16^. These approaches facilitated the proteomic analyses of filamentous fungi in the past decade^13^. In this study, SWATH acquisition method was applied to determine the differential proteins relating to sclerotia formation from hypha of *P. umbellatus*.

Sclerotia formation includes initial, developmental and mature phases, in which the initial phase is more important. Thus, we particularly focus on the proteomes of sclerotia and hyphae at initial phase to reveal the transition mechanisms, whereas the proteomes in developmental and mature phases were preliminary inspected. A number of proteins associating with OS generation, glycolysis induction as well as antioxidant activity were identified. To our knowledge, this is the first molecular evidence that antioxidant system is coordinated with OS development during *P. umbellatus* sclerotia formation to maintain cellular redox balance. The integrative assessment of *P. umbellatus* proteomes provides molecular bases for unveiling the sclerotia differentiation mechanism, which may also be applicable for other fungi.

## Results

### Global proteome analysis of *P. umbellatus*

**Figure 1:**
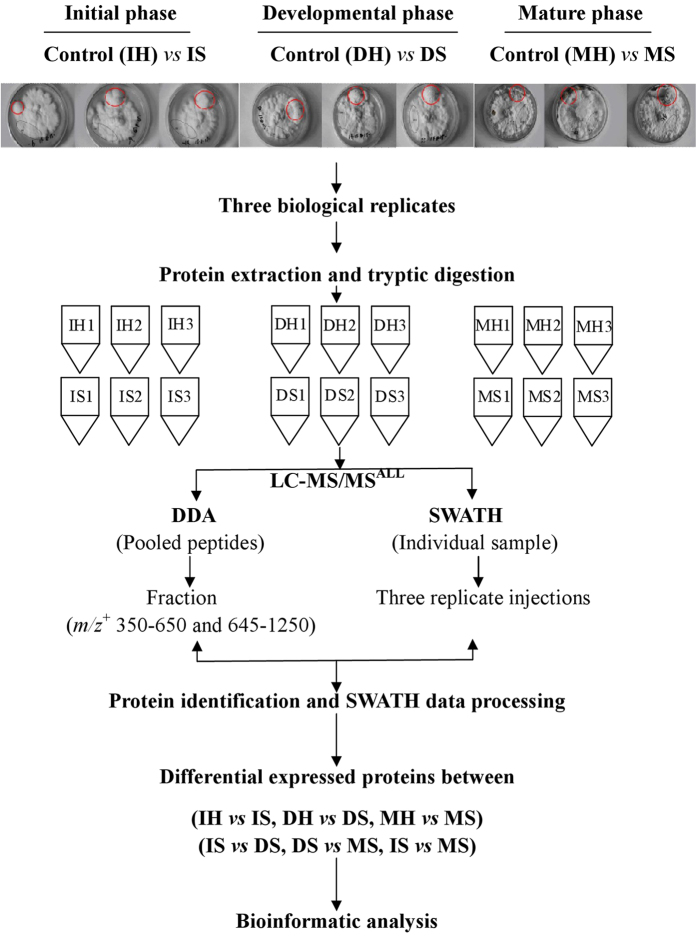
Scheme for *Polyporus umbellatus* proteomics. Red circle: sclerotia (S); black circle: hypha (H). I: initial; D: developmental; M: mature.

Considering temporal and spatial variation of proteomes, we chose sclerotia and hyphae of *P. umbellatus* in the same petri dish at initial, developmental and mature phases in triplicate as experimental specimen. The pooled and tryptic digested protein samples were analyzed by tandem LC-MS in data dependent acquisiation (DDA) model, and the acquired data were processed by Paragon^TM^ (AB Sciex) (Fig. 1). Overall, 1391 proteins in the pooled samples of *P. umbellatus* were identified with protein level at 1% and global false discovery rate (FDR) 63.7% (Supplemental Table S1), which represented the entire detectable proteins of *P. umbellatus* in both hypha and sclerotia covering the three growth phases. With SWATH-MS analysis, 1260 proteins were extracted from 54 SWATH data files, in which 1234 proteins were quantified.

**Figure 2:**
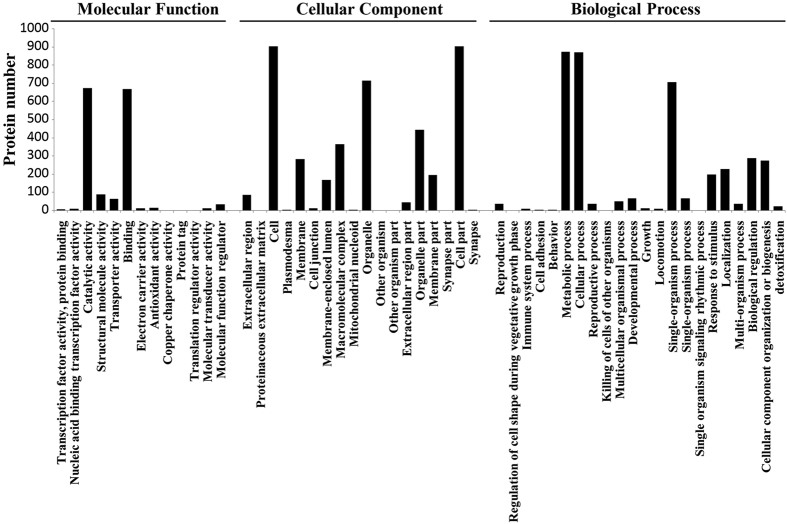
GO annotations of all quantified proteins.

The quantified proteins were classified into three major functional ontologies (cellular component, molecular function and biological process) by Gene Ontology (GO) enrichment analyses (Fig. 2). Most proteins located in cell, cell part, organelle, organelle part, macromolecular complex, membrane and membrane part, and some proteins located in extracellular region, cell junction and proteinaceous extracellular matrix. For molecular function, majority of proteins were assigned to catalytic activity, binding, structural molecule activity and transporter activity, but typical proteins participating electron carrier and antioxidant activity were also revealed, implying that oxidative stress might be developed in sclerotial differentiation and growth as a mechanism of cells responding to stimulus and detoxification. In biological process, the dominant subcategories were metabolic process, cellular process, single-organism process, biological regulation, cellular component organization or biogenesis and localization. Besides, proteins relating to ‘cell adhesion’ were also indicated.

Based on Kyoto Encyclopedia of Genes and Genomes (KEGG) metabolic pathway analysis (Supplemental Figure S1), the quantified proteins fell into five subcategories. In ‘Cellular Processes subcategories’, 66 proteins were involved in transport and catabolism, 44 involved in cell growth and death and 25 involved in cellular community. 134 proteins were associated with signal transduction of ‘Environmental Information Processing’. 113 and 75 proteins were involved in translation and folding, sorting and degradation of ‘Genetic Information Processing’ respectively. In ‘Metabolism subcategory’, large proportion of proteins was involved in carbohydrate metabolism (130), amino acid metabolism (112), energy metabolism (63) and lipid metabolism (40). There were seven proteins taking part in environmental adaption of ‘Organismal Systems’, indicating that they might play roles in triggering sclerotial differentiation from hyphae.

Global proteome analysis suggested that proteins in *P. umbellatus* are not only involved in essential primary metabolisms, but also associate with cell responses to external stimulus, such as oxidative stress and environmental adaption, which are significant for sclerotia initiation and development.

### Differentially expressed proteins in sclerotia and hyphae of *P. umbellatus*

**Figure 3:**
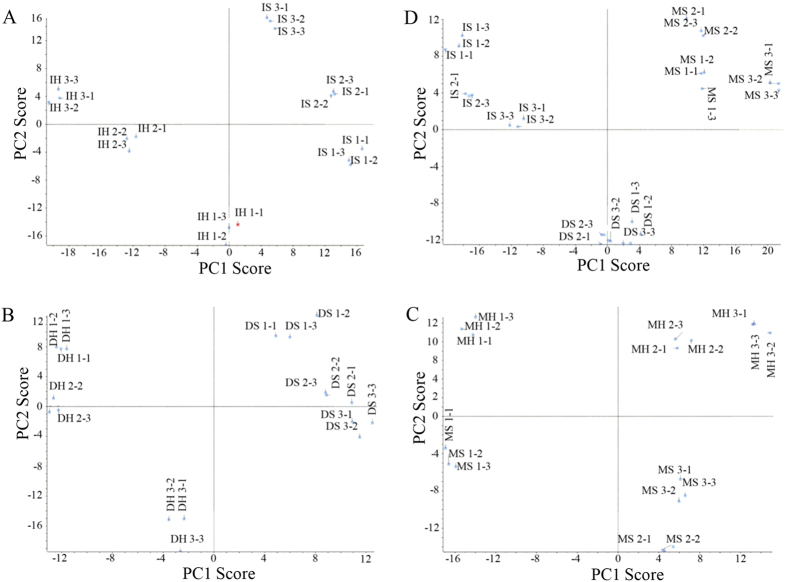
PCA score plots of proteome data in sclerotia and hyphae. PCA plots compared between sclerotia and hyphae were shown in (**A**) (IS and IH), (**B**) (DS and DH) and (**C**) (MS and MH), and among sclerotial proteomes at the three time points (**D**).

To understand how proteins were regulated during of *P. umbellatus* sclerotia formation from hyphae, quantitative proteomics were performed with label-free SWATH-MS to determine differentially expressed proteins (DEPs). Principal component analysis (PCA) showed that there was good reproducibility on the three injections of each sample and there were significant diversities between sclerotia and hyphae (Fig. 3). The proteins with significant differences in expression were obtained by T-test (p-value ≤ 0.05, fold change ≥1.5 or ≤0.667) between sclerotia and hyphae (or sclerotia) at initial, developmental and mature phases.

**Figure 4:**
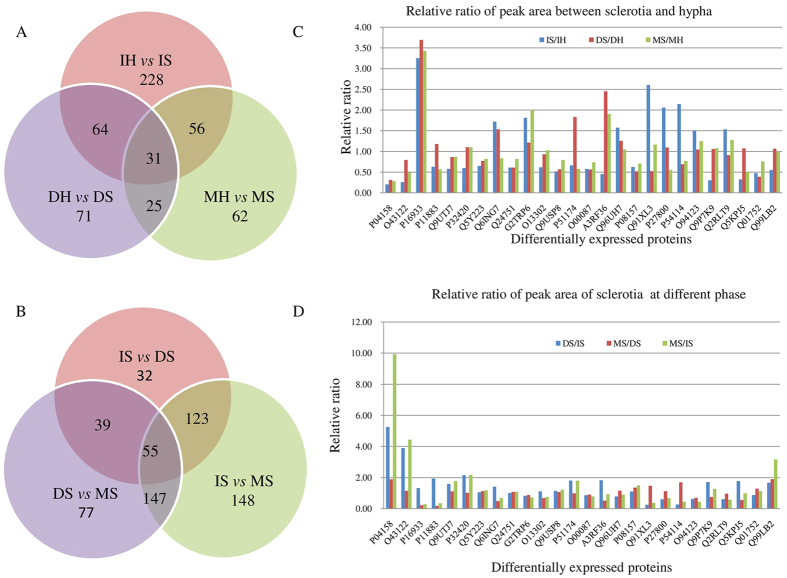
Venn diagram and the relative ratio of peak area of differentially expressed proteins in *P. umbellatus* sclerotia and hyphae. (**A**) Venn diagram of differentially expressed proteins between sclerotia and hyphae at initial, developmental and mature phases. (**B**) Venn diagram of differentially expressed proteins in sclerotia at initial, developmental and mature phases. (**C**) relative ratio of peak area of representative differentially expressed proteins between sclerotia and hyphae at initial, developmental and mature phases. (**D**) relative ratio of peak area of representative differentially expressed proteins in sclerotia at initial, developmental and mature phases.

At initial phase, 378 proteins were expressed differentially in sclerotia compared with those in hyphae including 322 identified proteins, and 56 unknown proteins that were labeled as “comp”. At developmental and mature phases, the amount of differential proteins decreased to 191 (28 unknown proteins and 163 identified proteins) (DS *vs* DH), and 174 (35 unknown and 139 identified proteins) (MS *vs* MH) respectively. 31 proteins were expressed differentially in sclerotia at all the three phases as shown in the Venn diagram (Fig. 4A), indicating that these proteins may be more indispensable in sclerotia formation.

During sclerotia growth, proein profiles changed in the two later phases (Fig. 4B). 249 proteins were expressed differentially in DS compared with those in IS including 36 unknown proteins and 213 identified proteins, and 319 differential proteins in MS compared with those in DS. Strikingly, nearly half of quantified proteins were expressed differentially in MS compared with those in IS suggesting that the cellular functions may be modulated substantially in mature phase. Moreover, sustained differential expression of 55 proteins in DS and MS relative to IS was observed as shown in the intersections of Venn diagram (Fig. 4B). It can be conceived that these DEPs may play essential roles during sclerotia growth for sclerotia development, maturation and the biomass increase following initiation.

#### GO analyses of DEPs associating with oxidative stress in sclerotia

Preliminary DEP analyses revealed that sclerotia formation in *P. umbellatus* could be multifactorial at initial, developmental and mature phases. To understanding what drive the transition from hyphae to scleorotia, GO analyses were performed on DEPs at initial phase.

**Figure 5:**
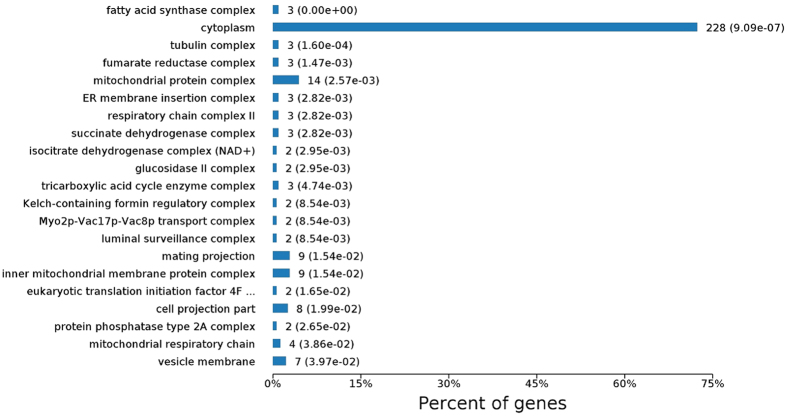
Cellular component of GO analyses on differentially expressed proteins between sclerotia and hyphae at initial phase.

In Cellular Component term of GO analysis, apart from the dominant proteins (228) located in cellular cytoplasm responsible for basic cellular activities, most of other DEPs were found in mitochondria (Fig. 5). There were 14 proteins in mitochondrial protein complex, 9 in the inner mitochondrial membrane protein complex, 4 in mitochondrial respiratory chain and 3 in respiratory chain complex II. Among them, respiratory chain-related protein SDHA (succinate dehydrogenase [ubiquinone] flavor protein subunit (FP), Q9UTJ7) and SDHB (succinate dehydrogenase [ubiquinone] iron-sulfur subunit (IP), P32420) of complex II, ATP synthase subunit beta (Q24751) of complex V, and Alpha-ETF (electron transfer flavor protein subunit alpha, Q5Y223) were down regulated in IS to 0.58, 0.60, 0.61 and 0.65 folds relative to IH, respectively. Flavin adenine dinucleotide (FAD) synthase (Q6ING7) and cytochrome c oxidase subunit 6B-like protein (G2TRP6) in sclerotia were increased to 1.72 and 1.81 folds, respectively. In addition, some proteins involved in tricarboxylic acid cycle (TCA) were expressed differentially, such as subunits of fumarate reductase complex, isocitrate dehydrogenase complex (NAD+), succinated dehydrogenase complex, and TCA enzyme complex. Isocitrate dehydrogenase subunit1 (IDH1, O13302) and subunit 2 (IDH2, Q9USP8), and long-chain specific acyl-CoA dehydrogenase (P51174) were expressed at a ratio of 0.62, 0.52 and 0.66 in sclerotia relative to those in hyphae, respectively. During sclerotia growth, SDHA and SDHB were increased in DS compared with those in IS, but there was no difference between MS and DS. Cytochrome c oxidase subunit 6B-like protein (G2TRP6) was consistently up-regulated in sclerotia at the three phases. Long-chain specific acyl-CoA dehydrogenase was increased with sclerotia growth and the relative ratio reached to 1.83 at developmental phase in DS compared with that in DH (Fig. 4C,D and Supplementary Table S2).

From Cellular Component ontology analysis, it is indicated that the respiratory chain reaction and TCA cycle (Fig. 5) as well as antioxidant system were modulated during sclerotia initiation, which were further evidenced in Biological Process and Molecular Functions subcategories (Supplementary Figures S2 and S3). 72 differential proteins taking part in ‘oxidation-reduction processes (Fig. S3), and proteins involved in the ‘response to stimulus’ were expressed differentially in IS compared with those in IH. Oxidoreductase activities acting on ‘CH-CH group’, ‘aldehyde or oxo group’, ‘paired donors’, ‘NAD(P)H’, and ‘sulfur group of donors’ were characterized (Fig. S2). Thus, GO analysis revealed that a number of proteins involved in oxidative stress and antioxidant system were differentially expressed, suggesting their fundamental roles in the transition of hyphae to sclerotia.

#### KEGG pathway analysis of DEPs associating with oxidative stress in sclerotia

To determine the pathways that DEPs may participate during sclerotia generation of *P. umbellatus*, KEGG analysis were carried out on the protomes at initial phase (Supplementary Figure S4). Apart from dominant DEPs assigned to secondary metabolic pathways, some proteins involved in TCA cycle, pyruvate metabolism and glycolysis/gluconeogenesis were expressed differentially, suggesting that *P. umbellatus* may encounter hypoxia before sclerotia differentiation^17^,^18^. Phosphoglycerate kinase (O94123) and 2, 3-bisphosphoglycerate-independent phosphoglycerate mutase (Q2RLT9) involved in glycolysis were up-regulated in IS, but decreased in DS and MS with sclerotia growth (Fig. 4C,D and Supplementary Table S2). Dihydrolipoyl dehydrogenase, pyruvate dehydrogenase-like protein, was decreased in sclerotia compared with hypha at initial phase, whereas various alcohol dehydrogenases and aldehyde dehydrogenases were up-regulated to generate NADH or NADPH (Fig. 4C,D and Supplementary Table S2), which is required for GSH biosynthesis to eliminate ROS under environmental stress. These results suggested that *P. umbellatus* might suffer from oxidative stress, which could induce sclerotia differentiation from hypha. Interestingly antioxidant system appeared to be activated which would be vital for maitaining redox balance.

#### Protein-protein interaction network in sclerotia formation

**Figure 6:**
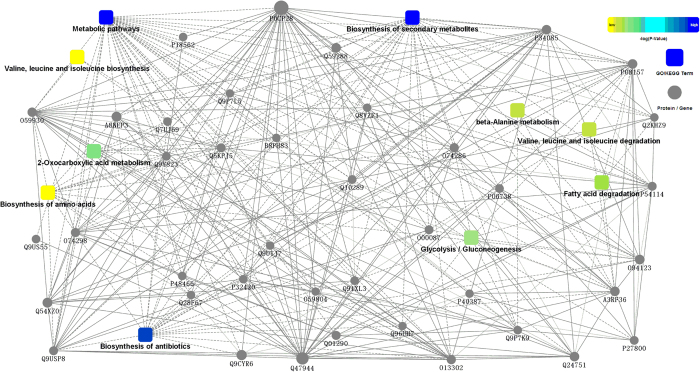
Protein-protein interactions network of differentially expressed proteins in *P. umbellatus* sclerotia and hyphae at initial phase.

To understand the relationships among proteins during the transition from hyphae to sclerotia, protein-protein interactions (PPI) were established by Omicsbean^TM^ on DEPs in sclerotia at initial phase (Fig. 6). There were 47 nodes (proteins) in the network. 10 GO/KEGG terms were indicated associating with ‘biosynthesis of antibiotics’, ‘glycolysis/gluconeogenesis’ and other metabolisms, such as ‘fatty acid degradation’, ‘2-oxocarboxylic acid metabolism’ and ‘biosynthesis of secondary metabolites’. In these terms, there were 22 proteins involved in ‘biosynthesis of antibiotics or detoxification’, 14 in ‘glycolysis/gluconeogenesis’, and 8 proteins producing NADH or NADPH (Fig. 6 and Supplementary Table S2) involved in anti-oxidant reactions. These proteins had connections with five terms and directly or indirectly interacted with other proteins. A3RF36 (aldehyde dehydrogenase) was not only involved in ‘biosynthesis of antibiotics or detoxification’ and ‘glycolysis/gluconeogenesis’, but also in ‘fatty acid degradation’, ‘beta-Alanine metabolism’ and ‘valine, leuline and isoleuline metabolism’. It had interactions with P32420 (SDHB) associating with metabolic pathways and respiratory chain. A3RF36 also had indirect (dot line) connections with Q5KPJ5 (acetolactate synthase), and direct connections (solid line) with other 10 proteins, such as O94123 (phosphoglycerate kinase). In addition, pyruvate dehydrogenase (O00087) was down regulated, indicating that reactions with O_2_ participation may be restrained in hypoxia based on the enzyme function. However, pyruvate kinase (PK, O94122), alcohol dehydrogenase and aldehyde dehydrogenase in glycolysis/gluconeogenesis were elevated. They are responsible for the synthesis of ATP and NAD(P)H, and NAD(P)H is required for GSH to eliminate ROS^19^ (Supplementary Table S2). The up-regulation of these enzymes suggested that glycolysis may be induced in cells and antioxidant-defensing system may be initiated concomitantly. Thus, by informatic analyses on the proteomes of *P. umbellatus* and DEPs at different growth phases, it was revealed that oxidative stress and antioxidant function may be induced in sclerotial differentiation and formation, in which glycolysis appears to be the hub of these two mechanisms.

### Glycerol induced sclerotial formation

**Figure 7:**
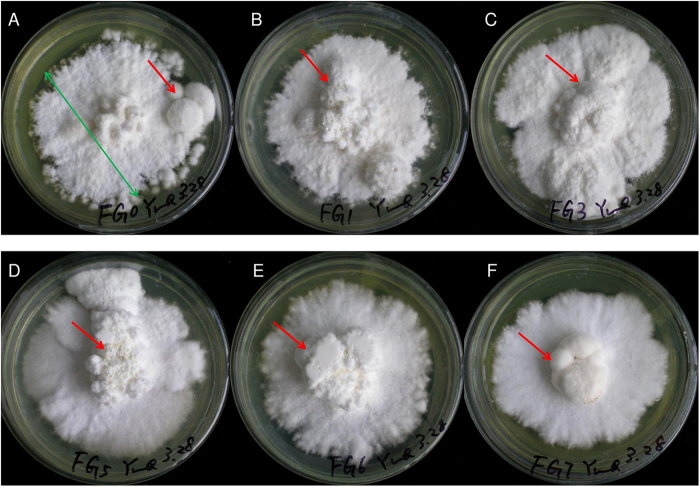
Glycerol induced and facilitated *P. umbellatus* sclerotia formation. (**A**) Sclerotia formed on fructose complete medium (control). (**B** to **F**) Sclerotia formed on fructose complete media containing 1% (**B**), 3% (**C**), 5% (**D**), 6% (**E**) and 7% (**F**) glycerol, respectively. The biomass of sclerotia was significantly increased at 5% glycerol compared with that on control medium (*p* < 0.05), and it was decreased at 6% and 7% glycerol. The colony diameter of mycelia (green arrow) was not affected by glycerol.

To verify the role of glycolysis in sclerotia induction, mimic assays with glycerol addition were carried out. It was shown that addition of 1% to 5% glycerol into fructose medium could induce sclerotia formation and facilitated mycelium and sclerotium growth. The colony diameters of mycelia were not significantly changed upon glycerol induction, but the fresh and dried weight of sclerotia were increased 157.9% and 313.3% respectively at 5% glycerol, which were significantly higher than those grew in fructose medium (Table 1 and Fig.7). However, the biomass was then reduced at higher glycerol concentration (6% and 7%), indicating that the induction of sclerotia by glycerol was concentration dependent.

To differentiate whether the impact of glycerol on sclerotia formation was due to glycolysis or osmotic pressure, KCl, NaCl, mannitol or sorbitol was added to the medium giving the same osmotic pressure as 5% glycerol. Sclerotia formation rate was 100% on fructose medium with 5% glycerol and 77.8% on control medium, and the dried weight were significantly increased 271.4% in glycerol containing medium. To our suprise, sclerotium formation was severely repressed by KCl, NaCl, mannitol or sorbitol (Table 2). These results demonstrated that glycerol inducing sclerotia formation was more likely due to glycolysis instead of osmotic pressure effect.

## Discussion

Sclerotium is a key growth stage in fungal life cycle, and confers fungi enhanced survivability and resistance to antibiotics. Pathogenic fungal sclerotia caused severe economic loss due to the difficulties tackling the sclerotia, whlie on the other hand, the threat of resource exhaustion on some valuable edible and medicinal fungal sclerotia, such as *P. umbellatus*, is emerging. Dissecting the formation mechanism would be helpful for rational control of sclerotia generation. Researches have been focused on the transition mechanism from hypha to sclerotia. Guo and his coworkers previously found that the growth and development of *P. umbellatus* sclerotia depended on symbiotic fungus, and the latter could induce *P. umbellatus* to generate reactive oxygen species (ROS) and enhance sclerotial formation^9^. There are a number of factors can result in ROS accumulation apart from symbiotic fungus. To make the proteomic analyses more achievable, *P. umbellatus* in this study was cultured on fructose complete medium without *Armillaria* spp. and the sclerotia were formed at optimal fructose concentration. Comprehensive proteome analyses of *P. umbellatus* were performed and DEPs were determined to reveal the key proteins responsible for OS generation as well as ROS elimination during sclerotial differentiation, and to uncover how these two mechanisms coordinately function in cells.

The repression of respiratory chain reaction and TCA cycle may lead to ROS generation and oxidative stress. OS can be developed by ROS accumulation in cells, and SDH and IDH are two of the key enzymes associated with oxidative stress. Dysfunction of SDH of complex II catalyzing succinate to fumarate caused succinate enrichment and ROS generation^20^,^21^,^22^. In *P. umbellatus*, succinate dehydrogenase subunits SDHA and SDHB were only decreased at initial phase in sclerotia then returned to the level as in hyphae at developmental and mature phases (Fig. 4C,D and Supplementary Table S2), indicating ROS may be generated during sclerotial initiation. IDH is NAD^+^-dependent enzyme and catalyzes NADH production, which plays major role in oxidative decarboxylation of isocitrate in TCA cycle^23^,^24^,^25^. Reduction in IDH1 and IDH2 could impair NADPH biosynthesis to increase the ratio of NADP^+^ to NADPH, which then could enhance ROS production^24^. In *P. umbellatus*, IDH1 (O13302) and IDH2 (Q9USP8) were down regulated (Fig. 4C,D and Supplementary Table S2) in initial sclerotia. Thus, it can be envisaged that the modulation on these enzymes would promote ROS and oxidative stress generation to induce sclerotial initiation.

In *P. umbellatus*, DEPs analyses indicated that electron transfer in respiratory chain may be restrained and ATP synthesis may be disrupted. FAD synthase responsible for the synthesis of cofactor FAD of complex II was increased nearly two folds in sclerotia at initial phase, and this could block electron transferring from reductase to oxygenase domain in respiratory chain^26^. Subunit beta (Q24751) of ATP synthase complex V and Alpha-ETF (electron transfer flavoprotein subunit alpha, Q5Y223) serving as specific electron acceptors were down-regulated. However, Cytochrome c oxidase subunit 6B-like protein of complex III and IV was increased at initial, developmental and mature phases in slcerotia which could direct proton crossing cell membrane and catalyze O_2_ reduction to form water^27^.

**Figure 8:**
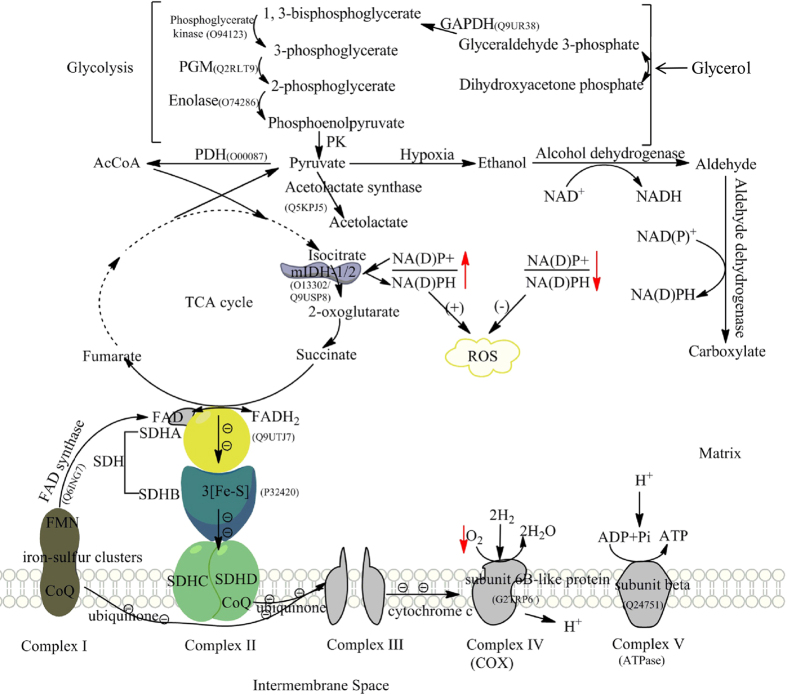
Proteins involved in ROS generation and elimination as a response to hypoxia in *P. umbellatus*. This schematic diagram was composed of electron transfer chain, TCA cycle and glycolysis. The expression of SDH subunits of complex II and IDH in TCA cycle was decreased in *P. umbellatus* sclerotia at initial phase leading to the increased ratio of NAD(P)+/NAD(P)H and ROS accumulation. NAD(P)H required for GSH to eliminate ROS could be accumulated with elevated alcohol dehydrogenase and aldehyde dehydrogenase following glycolysis/gluconeogenesis activation.

Glycolysis/gluconeogenesis pathway may be activated in sclerotial differentiation of *P. umbellatus*. It has been reported that decreased respiratory chain complexes of *S. cerevisiae* in response to hypoxia led to increased glycolysis to produce sufficient energy^24^,^25^. The product generated from glycolysis, pyruvate is the substrate of acyl-CoA in aerobic condition and the substrate of acetolactate, ethanol and aldehyde biosynthesis in hypoxia (Fig. 8). Dihydrolipoyl dehydrogenase, a subunit of mitochondrial pyruvate dehydrogenase (PDH) complex, could convert pyruvate either aerobically to acetyl-CoA or anaerobically to lactate^28^,^29^. Hypoxia-induced factor 1 (HIF-1) activated pyruvate dehydrogenase kinase 1 (PDK1) but inactivated PDH resulting in TCA cycle and mitochondrial respiration suppression, and activated other pathways generating ATP under hypoxia to keep tumor cells survival under hypoxia^13^,^30^. Dihydrolipoyl dehydrogenase (O00087) was down regulated in initial and developmental sclerotia of *P. umbellatus*, and the expressions of 2, 3-bisphosphoglycerate-independent phosphoglycerate mutase (Q2RLT9) and phosphoglycerate kinase (O94123) associated with glycolysis/gluconeogenesis were elevated in initial sclerotia. These data indicated TCA cycle and respiratory chain reaction may be repressed, and glycolysis may be induced, which can cause ROS and oxidative stress generation (Fig. 8).

Glycolysis associated with both oxidative stress and antioxidant defenses appeared to be a key mechanism in sclerotia formation. The impact on sclerotia initiation was investigated with glycerol addition. In the mimic assays, different concentration of glycerol in solid medium induced *P. umbellatus* sclerotia formation, and the sclerotia biomass was increased at the concentration lower than 5% (Fig. 7 and Table 1). Besides, sclerotia could be formed on the medium with 50 g/L fructose, but not on the medium that contained glucose or glycerol as single carbon resources^31^. Addition of glycerol into fructose medium could enhance sclerotial formation, which was not due to the osmotic pressure change. These results suggest that glycerol plays important roles in enhancing sclerotial formation. In addition, glycerol can be utilized as carbon resource through glycolysis pathway. Therefore, we concluded that glycerol induction on sclerotia generation was due to the activation of glycolysis but not osmotic pressure effect^32^ (Table 2), and glycolysis can be a hub for multiple redox balancing mechanisms.

Although oxidative stress is a key inducer of sclerotia, elimination of ROS efficiently is essential for its growth. GSH emerges as a main line to scavenge hydrogen peroxide or lipid hydroperoxide^19^. Higher ratio of NADPH to NADP is required for regeneration of glutathione (GSH) to resist oxidative stress in mitochondria, which could be increased by overproduction of dehydrogenase^33^,^34^. Thus, NADPH producer can execute defensing function against oxidative stress-induced damage, such as lipid peroxidation and concurrent mitochondrial damage with a significant reduction in ATP level^35^.

In glycolysis, pyruvate can be converted to ethanol and aldehyde under hypoxia, which then be catalyzed by alcohol dehydrogenase and aldehyde dehydrogenase to generate aldehyde or carboxylate respectively. NAD(P)H formed in this process, can result in increased NAD(P)H/NAD(P)^+^ ratio^36^ and GSH accumulation (Fig. 8). Acetolactate synthase is another enzyme in pyruvate biosynthesis and metabolism, participating L-isoleucine synthesis from 2-oxobutanoate and catalyzing the first step in valine biosynthesis with two pyruvate molecules conversion to one acetolactate molecule^37^,^38^. Up regulation of this enzyme in *Aspergillus nidulans* was correlated with NAD(P)H reduction for the synthesis of L-isoleucine or valine rapidly in response to hypoxia^39^. Acetolactate synthase (Q5KPJ5) and PDH in sclerotia of *P. umbellatus* were down regulated at initial phase, which may induce the pyruvate hypoxia metabolism to produce NADPH for GSH biosynthesis to eliminate ROS. Protein-protein interaction network (Fig. 6) established with DEPs of *P. umbellatus* sclerotia at initial phase revealed that there were various types of alcohol dehydrogenase and aldehyde dehydrogenase associated with glycolysis/gluconeogenesis. These proteins were increased in developmental or mature sclerotia, which would catalyze NADPH formation for GSH biosynthesis to exert antioxidant functions in DS and MS (Supplementary Table S2, Figs 4D and 7). Analdehyde reductase 1 (P27800) catalyzing alcohol to aldehyde and NADPH formation was increased 2.06 folds in sclerotia at initial phase, indicating its antioxidant activity. It was also note worthy that not all proteins (P08157, P11883, Q9P7K9 etc.) relating to NADPH and GSH generation were increased at the same time, implying the complexity of antioxidant system in cells. Another mechanism cell eliminating ROS is associated with long-chain specific acyl-CoA dehydrogenase. It was involved in the initial step of mitochondrial beta-oxidation of straight-chain fatty acids and in the maintenance of an internal steady state of lipid within an organism or cell^40^. This enzyme (P51174) in *P. umbellatus* sclerotia was reduced at the initial phase, but with sclerotia growth and mature, its expression was gradually increased to 1.83 folds at developmental phase in DS to DH. Thus, long-chain specific acyl-CoA dehydrogenase was modulated dynamically with sclerotia maturation to assure the frequency, rate or extent of ROS generation. Mitochondrial membrane lipid and cells could be prevented from ROS damage via enhancing long-chain specific acyl-CoA dehydrogenase expression. From these results, it can be envisaged that antioxidant activity was precisely controlled at each growth phase, and proteins involved in this function could be various from phase to phase.

Apart from DEPs associated with oxidative stress development and antioxidant function, the expression of some other proteins in sclerotia from initial to mature phase were changed significantly. Three hydrophobin proteins were revealed, and SC1 and B were decreased in sclerotia while SC3 was constantly high than that in hypha. Hydrophobin is a type of adhesion-like wall proteins secreted by hyphae and play essential role in association and adhesion fungal hypha to hydrophobic surfaces. Protein rds1 (P53693) was reduced to 0.22 folds but increased to 1.6 to 1.7 folds in developmental and mature sclerotia compared with that in hyphae, and strikingly, its expression in MS was 18.3 folds of that in IS. The functions of these proteins need to be further investigated.

## Conclusion

Sclerotium is a special form of many species of fungi with compacted hypha and enhanced survivability. Dissection the mechanism of sclerotia formation would be beneficial for resource revival of medicinal fungi and for fungal pathogen control. Quantitative proteome analysis in hypha and sclerotium of *P. umbellatus* at initial, developmental and mature phases were performed and DEPs were determined. GO annotation, KEGG pathways and Protein-Protein Interaction analyses on DEPs showed that oxidative stress played essential roles in triggering sclerotia differentiation from hypha of *P. umbellatus*, whereas antioxidant activity associated with glycolysis was critical for redox balancing. Oxidative stress may be developed due to the decrease of succinate dehydrogenase subunit of complex II in respiratory chain, isocitrate dehydrogenase in TCA cycle and dihydrolipoyl dehydrogenase, and the increase of FAD synthase, which may cause respiratory chain and TCA cycle disruption leading to glycolysis/gluconegenesis activation. Meanwhile, enzymes enhancing NAD(P)H and GSH production were revealed to eliminate ROS. Alcohol dehydrogenase and aldehyde dehydrogenase, and long-chain specific acyl-CoA dehydrogenase as well as other NAD(P)H producer exerting anti-oxidative functions were elevated. Thus, antioxidant defenses are concomitant with oxidative stress development in *P. umbellatus* sclerotia generation. Proteins revealed in this study by proteomics analyses associated with redox balancing provided new insights into sclerotial differentiation from hyphae in *P. umbellatus*, which may also be applicable for other fungi.

## Methods

### Strains and culture conditions

*P. umbellatus* isolated from the wild triennial sclerotia was rejuvenated and cultured as previously described^10^. *P. umbellatus* was inoculated on plates containing fructose complete medium (fructose 50.0 g/L, MgSO_4_·7H_2_O 0.5 g/L, KH_2_PO_4_ 0.5 g/L, vitamin B1 0.05 mg/L and agar 10.0 g/L) and cultured at room temperature in dark.

### Sclerotial and hyphal samples preparation

The procedures for sample sclerotia and hyphae collection and protein analysis were shown in Fig. 1. Sclerotia and hyphae collected on day 30 (initial phase), 40 (developmental phase) and 90 (mature phase) from the same plate were defined as IS, DS and MS respectively for sclerotia, and IH, DH and MH for hyphae as references. The sample mass was weighed and then frozen in liquid nitrogen until use. Three biological replicates were collected at each time point. The frozen hyphae and scleratia were ground in liquid nitrogen, and 100 mg of the ground materials were added with 300 ml protein extraction solution mix (BestBio Co. Ltd.) for filamentous fungi, containing solution A (protein extraction solution) and solution B (protease inhibitor) at a ratio of A to B 500:1 (*v*/*v*). The mixture was further ground to fine and transferred into Protein LoBind Tubes (Eppendorf, 1.5 ml). After incubated at 4 °C for 20 min at 150 rpm, the samples were centrifuged at 14000 rpm for 15 min, and total protein content in the supernatant was measured using BCA Protein assay kit (Beyotime Co. Ltd). The crude protein extracts (about 110 μg protein) were added with 20% trichloroacetic acid (TCA) to a final concentration of 10%, and precipitated for 3 h at −80 °C after vortex mix; then the protein pellets were collected by centrifugation at 20,000× *g* for 30 min at 4 °C, and re-dissolved homogenously in 200 μL ice cold acetone by ultra-sonication followed by incubation at −80 °C for 1 h and centrifugation at 20,000× *g* for 30 min at 4 °C. Proteins in the supernatant were re-precipitated at −80 °C overnight and collected by centrifugation at 20,000× *g* for 30 min at 4 °C, and then dehydrated by acetone at room temperature (Note: do not over dry the sample). 70 μL 0.2% RapiGestSF^TM^ (Waters) in 50 mM ammonium bicarbonate (ABC) was added to fully dissolve the pellet by sonication. The samples were incubated for 10 min at 37 °C with shaking at 300 rpm with tube lid open to allow acetone evaporation. Then they were incubated in 50 mM ABC containing 10 mM Tris (2-carboxyethyl) phosphine (TCEP, BOC Sciences) at 60 °C for 30 min, and 10 mM iodoacetamide (IAA, Sigma) in 50 mM ABC in dark for 30 min at RT successively, and then treated with 2.5 μg Sequencing Grade Modified Trypsin (enzyme to protein ratio about 1:40) at 37 °C overnight to generate protein tryptic fragments. Then 5 μL 10% trifluoroacetic acid (TFA, Sigma) was added and the samples were incubated at 37 °C for 1 h followed by centrifugation at 20,000× *g* for 10 min at 4 °C to get rid of un-digested proteins and the supernatant containing tryptic peptides were filtrated with 10 kDa filter membrane (Sartorius) to remove peptides larger than 10 kDa. The final protein concentration was about 1 μg/μl. These tryptic peptides were stored at −80 °C until use^15^.

### Shotgun proteomic measurement

Peptides of all samples were pooled and measured by AB Sciex 5600+ TripleTOF platform configured with Eksigent 400nano-HPLC system (Eksigent, AB Sciex, U.S.) and 5600^+^ TripleTOF mass spectrometer (AB Sciex). Pooled peptides were desalted with a Nano LC loading trap (Eksigent, AB Sciex, 350 μm × 0.5 mm, Chrom XP C18-3 μm, 120 Å) by isocratic elution with solvent A (water containing 0.1% (v/v) formic acid and 2% (v/v) acetonitrile) at a flow rate of 2 μL min^−1^ for 10 min. Peptides were then separated on analytical column (75 μm × 20 cm, Sunchrom C18-5 μm, 120 Å, Beijing Happy Science Scientific Co. Ltd) by gradient elution with solvent A and B at a flow rate of 300 nL min^−1^. The gradient change is as follows: phase B (acetonitrile containing 0.1% formic acid and 2% water) was increased to 17% from 5% in 60 min, then changed to 27% in 25 min, and finally reached 50% in 10 min.

MS1 spectra were collected in high-resolution model in the range of *m*/*z* 350–650 and *m*/*z* 645–1250 respectively. TOF MS parameters were set as follows: Nano Spray; ion source gas 1(GS1) 10; curtain gas (CUR) 30; ion spray voltage floating (ISDF) 2300; interface heater temperature (IHF) 100; declustering potential (DP) 100; collision energy (CE) 10; accumulation time 250 ms; mass tolerance 50 mDa; exclude former target ions for 15 s after 1 occurrence. The 30 most intense precursors with charge state 2 to 5, which exceeded 200 counts per second (cps), were selected for fragmentation. MS2 spectra were collected in the range of *m*/*z* 100–1500 for 100 ms in high sensitivity model.

### Proteomic analysis with SWATH–MS

At first, pooled peptides spectra were acquired with the nano-LC MS/MS method as shown in DDA measurement, but TOF MS rang was set from *m*/*z*350 to 1250, and variable window for SWATH-MS was calculated using Variable Window Calculator V 0.2 112513 (AB Sciex). The parameters were set as follows: number of window 60; lower *m*/*z* limit 400; upper *m*/*z* limit 1250; window overlap (Da) 0.5; rolling collision energy (CES) 15. The effective isolation windows were considered as 400–420.5, 419.5–428.5, 437.5–451.5 etc. MS2 spectra were collected in the range of *m*/*z*100 to1500 for 40 ms in high sensitivity model and the resulting total cycle time was 1.42 s. CES 15 v, charge state 2 and CE 10 v were applied. SWATH-MS measurements were performed in triplicate for each sample.

### Data processing and bioinformatics analysis

The pooled peptide data acquired in DDA model were analyzed by Paragon (ProteinPilot software, AB/Sciex, v. 5.0.0.0, 4769) against “zhuling_uniprot_full. fasta” database which was build based on transcriptome from our own lab of *P. umbellatus* (zhuling)^41^. There were 22936 proteins in this database (.fasta). Settings for Paragon search were as follows: Sample type: Identification; Cys Alkylation: Iodoacetamide; Digestion: Trypsin; Instrument: TripleTOF5600; Special Factors: no; Species: none; Search effort: Thorough ID; Detected proteins threshold (unused Protscope (conf)) of Results Quality: 0.05 (10%), and false discovery rate (FDR) analysis was chosen.

An ion library was generated from data acquired in DDA model for targeted extraction in SWATH model. Linear regressions were conducted on the retention time (RT) from different data sources between DDA data and the SWATH data using PeakView SWATH Processing Micro App (AB Sciex, v.1.0.0.1409). All of the RTs of the identified peptides from DDA were globally corrected resulting in a new ion library consisting of the peptides with corrected RTs. The parameters for processing SWATH data were as follows: maximum number of proteins to import 3000; number of peptides per protein 6; number of transitions per peptide 6; peptide confidence threshold 95%; FDR threshold 1.0%; 50 ppm *m*/*z* tolerance; 15 min extraction window; shared peptides excluded for SWATH analysis; and modified peptides included.

Protein peak area data were exported and processed with Markerview (AB SCIEX, 1.2.1.1). Principle component analysis (PCA) and T-test were performed for IH *vs*. IS, DH *vs*. DS, MH *vs*. MS, IS *vs*. DS, DS *vs*. MS and IS *vs*. MS in which fold change was set as ≥1.5 or ≤0.067, and *p*-value less than 0.05 was chosen after area normalization. Differential proteins were picked out based on the above settings, and subjected to bioinformatics analysis. Then they were annotated by Gene Ontology Consortium (GO)^42^, and their systematic information was computed by Kyoto Encyclopedia of Genes and Genomes (KEGG)^43^. Differential proteins were imported into Omicsbean^TM^ to seek candidate proteins and their interaction network.

### Induction of P. umbellatus sclerotia by glycerol

Different concentrations (1%, 2%, 3%, 4%, 5%, 6% or 7%) of glycerol were added into fructose complete medium (glycerol-fructose medium). To evaluate the osmotic pressure effect, KCl, NaCl, mannitol and sorbitol (osmotic pressure regulator) were added into fructose complete medium (osmotic pressure regulator-fructose medium) to give the same osmotic pressure (988 mOsmol/kg) as 5% glycerol. Hyphae of *P. umbellatus* were inoculated onto these media and cultured as described above. On day 40, sclerotia were collected and the mecylial colony diameter and sclerotia biomass were measured.

## Additional Information

**How to cite this article**: Li, B. *et al*. SWATH label-free proteomics analyses revealed the roles of oxidative stress and antioxidant defensing system in sclerotia formation of *Polyporus umbellatus.* *Sci. Rep.* **7**, 41283; doi: 10.1038/srep41283 (2017).

**Publisher's note:** Springer Nature remains neutral with regard to jurisdictional claims in published maps and institutional affiliations.

## Supplementary Material

Supplemental Table S1

Supplemental Table S2 and Figures S1-4

## Figures and Tables

**Table 1:** Parameters of mycelium and sclerotia of *P. umbellatus* after glycerol addition.

Glycerol (%)	Colony diameterof mycelia (M ± SD) (cm)	Fresh weight of sclerotia (M ± SD) (g/dish)	Dry weight of sclerotia (M ± SD) (g/dish)
0	6.96 ± 0.34 cd	1.14 ± 0.63a	0.15 ± 0.08a
1	7.27 ± 0.36d	2.02 ± 0.56ab	0.28 ± 0.08ab
2	6.84 ± 0.37c	1.86 ± 1.20ab	0.28 ± 0.17ab
3	6.81 ± 0.35c	2.11 ± 0.72bc	0.33 ± 0.08b
4	6.96 ± 0.19 cd	2.09 ± 1.14bc	0.38 ± 0.17b
5	7.06 ± 0.23 cd	2.94 ± 0.79c	0.62 ± 0.13c
6	6.32 ± 0.24b	1.83 ± 0.97ab	0.39 ± 0.18b
7	5.71 ± 0.59a	1.20 ± 0.58ab	0.28 ± 0.12ab

Note: the experiments were done in ten replicates (n = 10). Same letter (a, b, c, or d) indicatesthat there was no significant difference (P < 0.05) between these groups. M: mean, SD: standard deviation.

**Table 2:** Parameters of mycelium and sclerotia after different osmotic pressure regulator addition.

Osmotic pressure regulators	Colony diameterof mycelia (M ± SD) (cm)	Sclerotial formation rate (%)	Fresh weight of sclerotia (M ± SD) (g/dish)	Dry weight of sclerotia (M ± SD) (g/dish)
None	7.09 ± 0.33d	77.8	0.83 ± 0.65a	0.14 ± 0.11a
Glycerol	7.25 ± 0.18d	100	1.89 ± 0.61b	0.52 ± 0.09b
NaCl	3.96 ± 0.39a	0	—	—
KCl	5.77 ± 0.26c	0	—	—
Mannitol	5.70 ± 0.35c	0	—	—
Sorbitol	4.57 ± 0.53b	0	—	—

Note: the experiments were done in ten replicates (n = 10). Same letter (a, b, c, or d) indicatesthat there was no significant difference (P < 0.05) between these groups. “—” represents no sclerotia formation. M: mean, SD: standard deviation.
