# Supplementary material for: SWATH label-free proteomics analyses revealed the roles of oxidative stress and antioxidant defensing system in sclerotia formation of Polyporus umbellatus
Source: Sci Rep. 2017 Jan 30;7:41283. doi: 10.1038/srep41283 (PMC5278369; doi:10.1038/srep41283)
Supplement: Supplemental Table S2 and Figures S1-4 [file srep41283-s2.pdf]

SWATH label-free proteomics analyses revealed the roles of oxidative stress and antioxidant defensing system in sclerotia formation of *Polyporus umbellatus*

Bing Li[1], Xiaofang Tian[2], Chunlan Wang[1], Xu Zeng[1], Yongmei Xing[1], Hong Ling[1], Wanqiang Yin[3], Lixia Tian[1], Zhixia Meng[1], Jihui Zhang[4, *], Shunxing Guo[1, **]

(1. Institute of Medicinal Plant Development, Chinese Academy of Medical Sciences & Peking Union Medical College, Beijing 100193 P. R. China

2. Pharmaceutical department of China-Japan Friendship Hospital, Beijing 100029 P. R. China

3. Tianjin University of Science & Technology, Tianjin 300457, P. R. China

4. State Key Laboratory of Microbial Resources, Institute of Microbiology, Chinese Academy of Sciences, Beijing 100101, P. R. China)

Table S2. Proteins information and relative ratio of peak area that related with oxidative stress, glycolysis and cell wall adhesion

ID		Name	Catalytic funtion	Relative ratio of area					
				IS/IH	DS/DH	MS/MH	DS/IS	MS/DS	MS/IS
Respirotary chain	Q9UTJ7	succinate dehydrogenase (SDH) [ubiquinone] flavoprotein subunit (FP)	Succinate + a quinone = fumarate + a quinol	0.58	-	-	1.59	-	1.77
	P32420	Succinate dehydrogenase (SDH) [ubiquinone] iron-sulfur subunit	Succinate + a quinone = fumarate + a quinol	0.60	-	-	2.15	-	2.18
	Q5Y223	electron transfer flavoprotein subunit alpha	serves as a specific electron acceptor for several dehydrogenases	0.65	-	-	-	-	-
	Q6ING7	FAD synthase	ATP + FMN = diphosphate + FAD	1.72	1.53	-	1.41	0.49	-
	Q24751	ATP synthase subunit beta	ATP + H_2O + H^+(In) = ADP + phosphate + H^+(Out)	0.61	0.61	-	-	-	-
	G2TRP6	Cytochrome c oxidase subunit 6B-like protein	4 ferrocytochrome c + O_2+ 4H+ = 4 ferricytochrome c + 2 H_2O	1.81	1.21	2.00	-	-	-
TCA cycle	O13302	Isocitrate dehydrogenase [NAD] subunit 1 (IDH1)	Isocitrate + NAD^+ = 2-oxoglutarate + CO_2 + NADH+H^+	0.62	-	-	-	-	-
	Q9USP8	Isocitrate dehydrogenase [NAD] subunit 2 (IDH2)	Isocitrate + NAD^+ = 2-oxoglutarate + CO_2 + NADH	0.52	0.56	-	-	-	-
	P51174	Long-chain specific acyl-CoA dehydrogenase	Long-chain-acyl-CoA + electron-transfer flavoprotein = long-chain-2,3-dehydroacyl-CoA + reduced electron-transfer flavoprotein	0.66	1.83	0.58	1.81	-	1.80
Glycolysis/	O00087	Dihydrolipoyl dehydrogenase	N(6)-(dihydrolipoyl)lysine +	0.58	0.56	-	-	-	-

gluconeogenesis or Biosynthesis of antibiotics			NAD^+ = protein N(6)-(lipoyl)lysine + NADH						
	Q5KPJ5	Acetolactate synthase	2-pyruvate = 2-acetolactate + CO_2	0.32	-	0.50	1.77	0.56	
	O94123	Phosphoglycerate kinase	ATP + 3-phospho-D-glycerate = ADP + 3-phospho-D-glyceroyl phosphate	1.50	-	-	0.63	-	0.44
	Q2RLT9	2, 3-bisphosphoglycerate-independent phosphoglycerate mutase	2-phospho-D-glycerate = 3-phospho-D-glycerate	1.53	-	-	0.61	-	0.58
	P08157	Aldehyde dehydrogenase	An aldehyde + NAD^+ + H_2O = a carboxylate + NADH	0.62	0.52	-	-	-	1.50
	Q96UH7	Fructose-bisphosphate aldolase 1	D-fructose 1, 6-bisphosphate = glycerone phosphate + D-glyceraldehyde 3-phosphate	1.58	-	-	-	-	-
	Q91XL3	UDP-glucuronic acid decarboxylase 1	UDP-D-glucuronate = UDP-D-xylose + CO_2	2.61	0.52	-	0.25	1.47	0.37
	P11883	Aldehyde dehydrogenase	An aldehyde + $NAD(P)^+$ + H_2O = a carboxylate + NAD(P)H	0.63	-	0.57	1.94	0.18	0.35
	P54114	Aldehyde dehydrogenase [NAD(P)+] 2	An aldehyde + NAD^+ + H_2O = a carboxylate + NADH	2.15	-	-	0.26	1.69	0.45
	Q9P7K9	Aldehyde dehydrogenase-like protein C21C3	2-phospho-D-glycerate = 3-phospho-D-glycerate	0.30	-	-	1.71	-	-
	P27800	Aldehyde reductase 1	An alcohol + $NADP^+$ = an aldehyde + $NADPH+H^+$	2.06	-	0.55	0.61	-	-
	Q01752	Aryl-alcohol dehydrogenase [NADP(+)]	An aromatic alcohol + $NADP^+$ = an aromatic aldehyde + NADPH	0.48	0.39	-	-	-	-
	Q99LB2	Dehydrogenase/reductase SDR family	an alcohol + NAD(P)+ = an	0.55	-	-	1.67	1.90	3.17

		member 4	aldehyde + NAD(P)H + H+						
	A3RF36	Aldehyde dehydrogenase	An aldehyde + $NAD(P)^+$ + H_2O = a carboxylate + NAD(P)H+ H^+	0.45	2.45	1.91	1.83	0.51	-
Cell adhesion	P04158	Hydrophobin SC1	Contributes to surface hydrophobicity	0.21	0.31	0.28	5.25	1.89	9.93
	O43122	Hydrophobin B	Contributes to the structural integrity of a cell wall	0.26	-	0.49	3.90	-	4.44
	P16933	Hydrophobin SC3	Contributes to surface hydrophobicity	3.25	3.70	3.43	1.32	0.22	0.29

Note: “-”- ratio was between 0.67 and 1.50, there was no significant difference

Supplemental Figures

Fig.S1

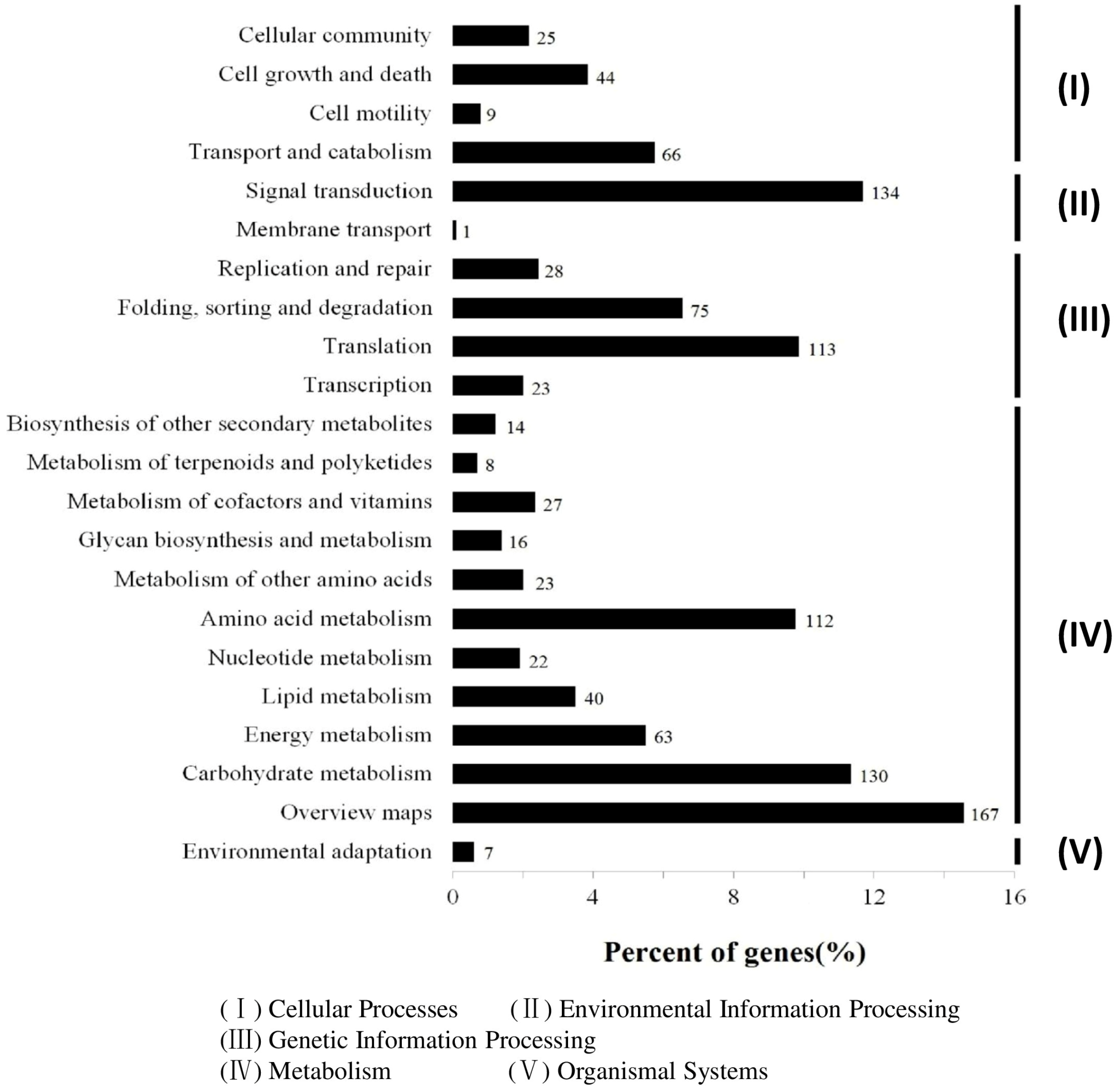

Fig.S1 KEGG metabolic pathway analyses of all quantified proteins.

Fig.S2

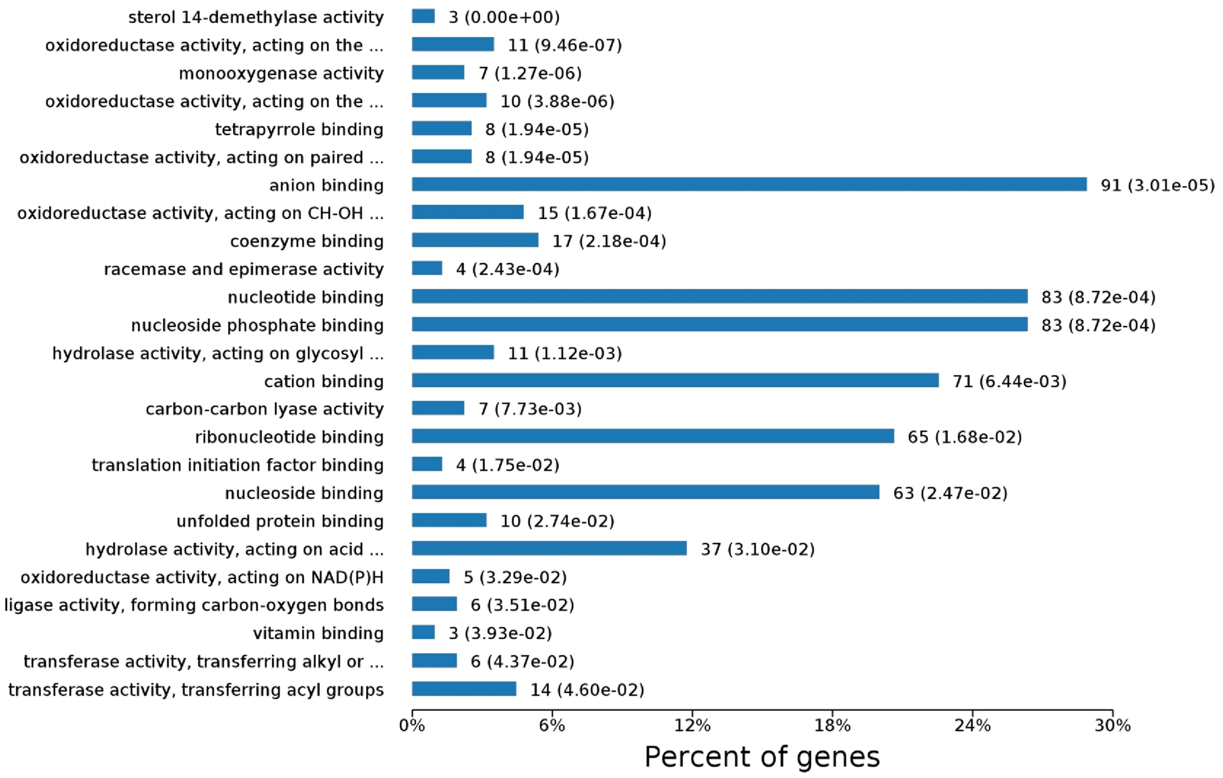

Fig.S2 Molecular Function of GO annotation for differentially expressed proteins between sclerotia and hyphae at initial phase.

Fig.S3

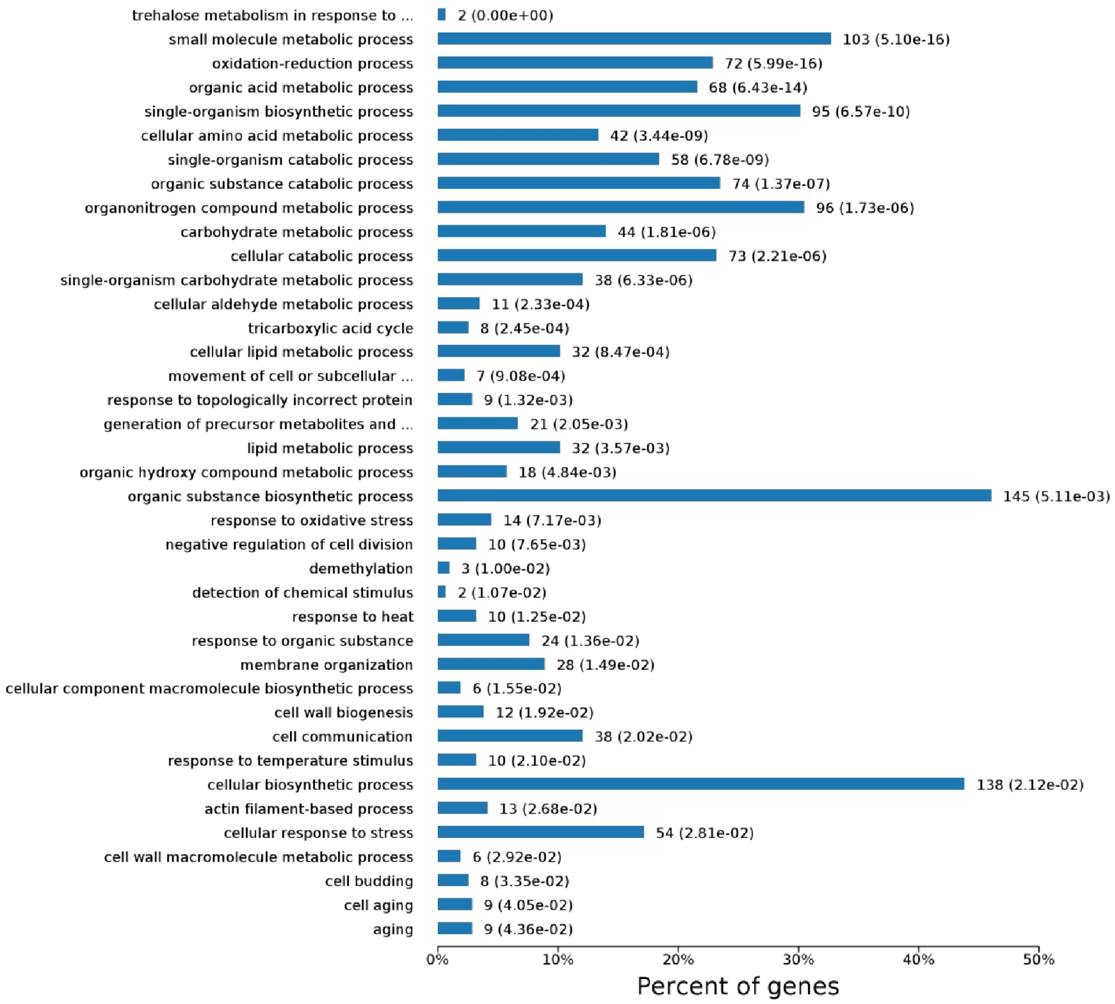

Fig.S3 Biological Process of GO annotation for differentially expressed proteins between sclerotia and hyphae at initial phase.

Fig.S4

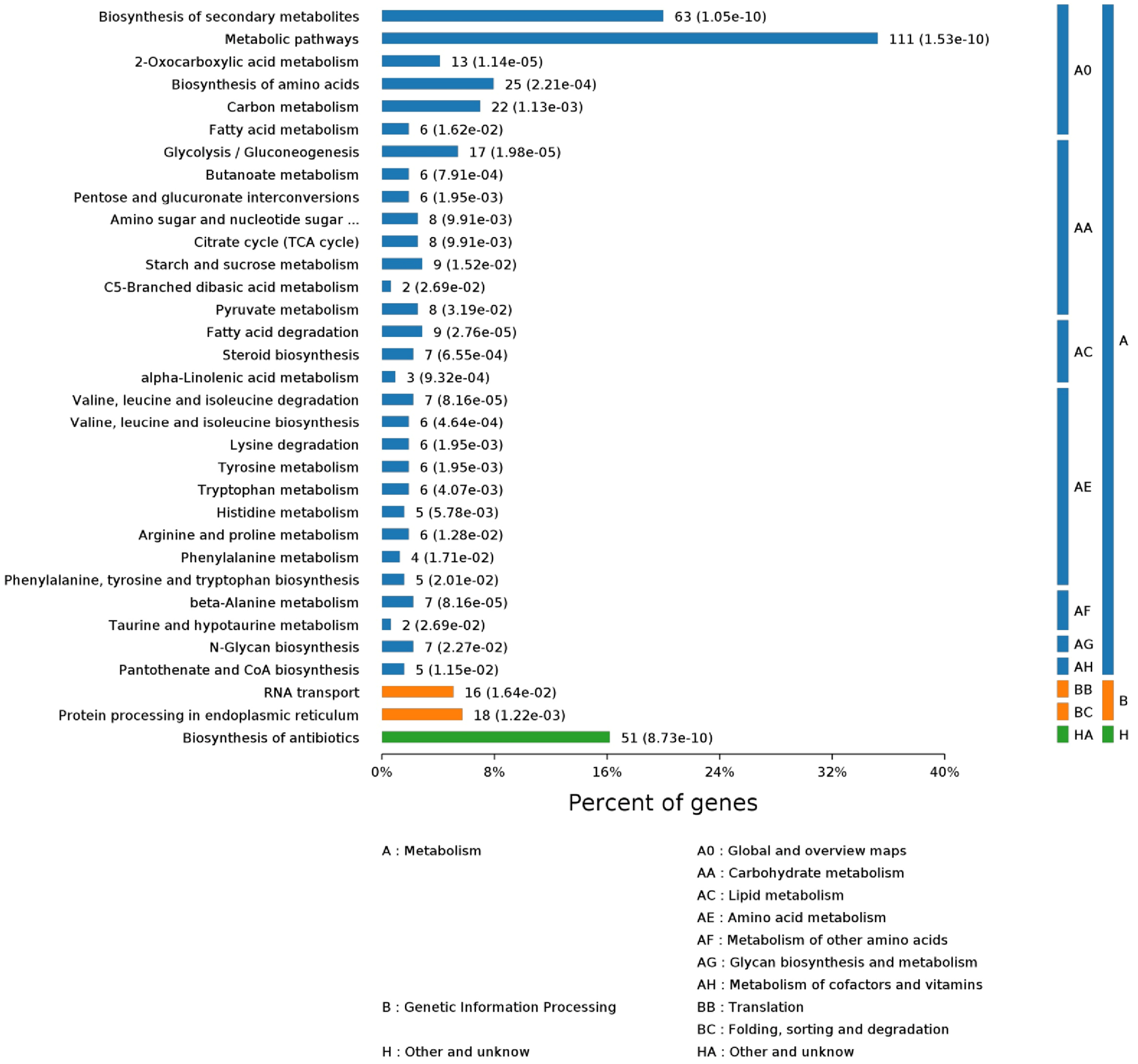

Fig.S4 KEGG metabolic pathway analyses of differentially expressed proteins between sclerotia and hyphae at initial phase.
